# Screening for postoperative vital signs abnormalities, and particularly hemodynamic ones, by continuous monitoring: protocol for the Biobeat-Postop cohort study

**DOI:** 10.12688/f1000research.54781.2

**Published:** 2021-10-07

**Authors:** Alexis Paternot, Philippe Aegerter, Aurélie Martin, Jonathan Ouattara, Sabrina Ma, Sherifa Adjavon, Bernard Trillat, Pascal Alfonsi, Marc Fischler, Morgan Le Guen

**Affiliations:** 1 Department of Anaesthesiology, Hopital Foch, Suresnes, 92150, France; 2 Methodology Unit, GIRCI-IdF, Paris, 75019, France; 3 Department of Research and Innovation, Hôpital Foch, Suresnes, 92150, France; 4 Department of Anaesthesiology, Groupe Hospitalier Paris Saint-Joseph, Paris, 75014, France; 5 Department of Information Systems, Hôpital Foch, Suresnes, 92150, France

**Keywords:** Surgery; Monitoring; Complications; Perioperative medicine

## Abstract

**Background**: Postoperative hypotension associated with postoperative morbidity and early mortality has been studied previously. Hypertension and other hemodynamic, respiratory, and temperature abnormalities have comparatively understudied during the first postoperative days.

**Methods**: This bi-centre observational cohort study will include 114 adult patients undergoing non-cardiac surgery hospitalized on an unmonitored general care floor and wearing a multi-signal wearable sensor, allowing remote monitoring ( Biobeat Technologies Ltd, Petah Tikva, Israel). The study will cover the first 72 hours after discharge of the patient from the post-anaesthesia care unit. Several thresholds will be used for each variable (arterial pressure, heart rate, respiratory rate, oxygen saturation, and skin temperature). Data obtained using the sensor will be compared to data obtained during the routine nurse follow-up. The primary outcome is hemodynamic abnormality. The secondary outcomes are postoperative respiratory and temperature abnormalities, artefacts and blank/null outputs from the wearable device, postoperative complications, and finally, the ease of use of the device. We hypothesize that remote monitoring will detect abnormalities in vital signs more often or more quickly than the detection by nurses’ routine surveillance.

**Discussion**: A demonstration of the ability of wireless sensors to outperform standard monitoring techniques paves the way for the creation of a loop which includes this monitoring mode, the automated creation of alerts, and the sending of these alerts to caregivers.

**Trial registration**: ClinicalTrials.gov, NCT04585178. Registered on October 14, 2020

## Introduction

Postoperative mortality remains a current controversial issue, as shown in 2016 by the International Surgical Outcomes Study. ^1^ This prospective international cohort study reported that 16.8% of patients developed one or more postoperative complications, and 0.5% died. Several studies have focused specifically on the risks of postoperative hypotension, ^2^ respiratory depression, ^3^ and hypoxemia. ^4^ This has been well demonstrated in particular with regard to unrecognized hypotension since the risk of myocardial ischemia is increased by cumulative durations of 2 to 4 h of hypotension (mean arterial pressure < 60 mmHg) or durations of more than 4 h with mean arterial pressure < 65 or 70 mmHg. ^5^

Most patients are hospitalized on an unmonitored general care floor (ward) where survival after a cardiac arrest is worse compared to ICU patients or patients hospitalized in a monitored ward setting. ^6^ However, postoperative placement in high-level monitoring units for patients with risk factors is impossible in view of their number. To limit the risk of missing an abnormality in one of the physiological parameters, the National Health Service in England proposed (imposed in fact) a rigorous and repeated evaluation of the clinical condition at regular intervals with early warning scores (EWS). ^7^ But this evolution in nursing practice does not prevent it from being spot check monitoring. Evolution of technology allows continuous and remote monitoring using either a bed-based mattress sensor, patient-worn monitor, and wearable patch sensors allowing continuous monitoring.

The most convenient system for patients, which makes them fully independent and therefore does not impede their mobility, is a skin patch that measures a wide range of vital signs at a frequent rate and automatically transmits this information to the nursing staff. Remote wireless vital sign monitoring on the ward has been reported in case series of medical or surgical patients ^8^ and more recently in patients suffering from COVID-19. ^9^

To promote the widespread use of these devices, it is necessary to confirm the comparative advantage of remote monitoring over conventional nursing monitoring. In the present study, we hypothesize that monitoring with a multi-signal wearable sensor will detect potentially dangerous vital sign abnormalities more often and more rapidly than routine surveillance in surgical patients hospitalized on an unmonitored general care floor. Data obtained using the sensor will be compared to data obtained during the routine nurse follow-up during at most 72 first postoperative hours.

## Methods

Ethics approval for this trial was obtained from the Ethical Committee Ile de France II (Paris, France) on September 28, 2020 (approval number: 2020-A01852-37) and registered at ClinicalTrials.gov under the trial identification number NCT04585178).

Written informed consent will be obtained by study staff from all study participants prior to their participation in study. Any modifications to the protocol will be sent to the Ethical Committee before they are implemented within the study and communication changes that impact the patients would require signing of a revised consent form. The current version is version n° 2; July 28; 2020. Complete protocol can be obtained on request. The study protocol has been reported in accordance with the Standard Protocol Items: Recommendation for Clinical Interventional Trials (SPIRIT) guidelines.

### Aim, study design and setting

This protocol seeks to quantify the benefit of remote monitoring using a multiparametric device in the detection of postoperative complications in comparison with the monitoring usually performed by nurses.

The Biobeat-Postop Protocol is a prospective observational bi-centre study that will be conducted in two private non-profit hospitals in which all types of surgical procedures, except cardiac procedures, are routinely performed. Patients will be consecutively enrolled and followed up for the first 72 postoperative hours after the patient leaves the post-anaesthesia care unit.

Recruitment of patients began on December 15, 2020, and is ongoing.

### Study population and eligibility criteria

Patients will be included if they meet the following criteria: (1) are over 18 years, (2) require general anaesthesia for a major surgical procedure (gastro-intestinal surgery, gynaecological surgery, urologic surgery, and orthopaedic surgery) with an expected duration of intervention of more than 2 hours, (3) need a planned postoperative stay of more than 72 hours, and (4) have provided written informed consent. The exclusion criteria will be any abnormality of the skin or a very hairy skin at the location of the patch, tremor, allergy to the components of the patch, planned scanner or magnetic resonance imaging during the postoperative course, and pregnancy. Consecutive patients will be screened unless the physician responsible for providing the patient information and consent is not available in accordance with the regulations.

### The studied device

**Figure 1.**
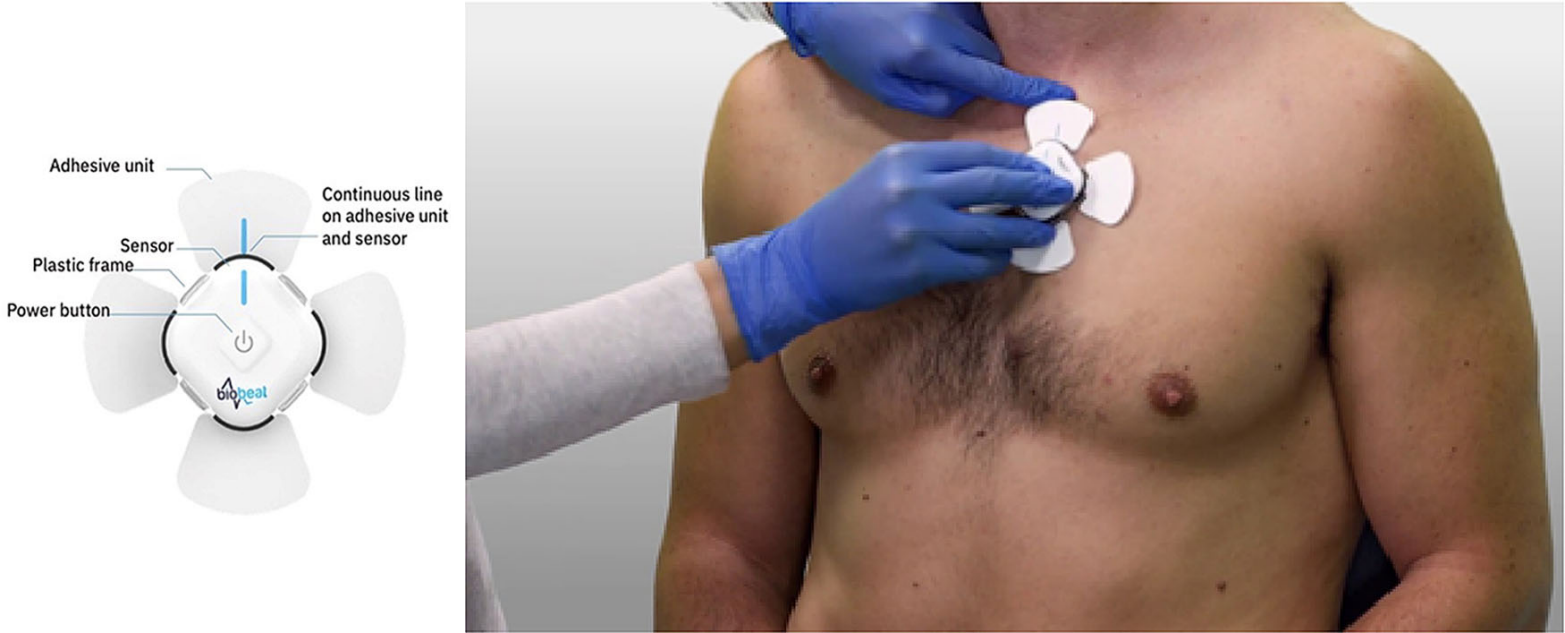
The sensor and placement of the patch sensor (consent obtained for publication).

Patients will be equipped with a portable Biobeat ^®^ sensor, a single-use patient device that consists of a skin patch placed 1 cm to the left of the sternum, just below the clavicle ( Biobeat Technologies Ltd, Petah Tikva, Israel) ( Figure 1). The sensor continuously records the photoplethysmographic waveform, which allows recording and calculation of several physiological parameters: heart rate (HR), respiratory rate (RR), peripheral oxygen saturation (SpO _2_), systolic arterial pressure (SAP) and SAP variation, diastolic arterial pressure (DAP) and DAP variation, and skin temperature. Other variables, stroke volume and cardiac output, are measured by this sensor but are not included in this protocol.

Biobeat ^®^ is CE approved (N°688840; March 19, 2019), and its use was also approved by the FDA in 2019 (510K clearance for measurement of arterial pressure, oxygenation and HR in hospitals, clinics, and long-term care and at home).

High agreement has been demonstrated for arterial pressure measurements obtained via the Biobeat ^®^ sensor and the gold-standard sphygmomanometer technique. ^10^

### Procedure

Patient selection will be performed during a preoperative anaesthesia consultation with one of the doctors on the research team. After verification of the inclusion and exclusion criteria, the physician will inform the patients about the study and obtain their written informed consent.

The patch will be put in place in the post-anaesthesia care unit just before the patient's return to the conventional hospitalization service. The monitoring data from the patch will be recorded continuously without being communicated to the nursing staff and doctors.

Postoperative medical, surgical, and nursing care will be in accordance with medical indications depending on the clinical routine, without any study-specific restrictions, and all the nurses’ observations (clinical observations and measurements) will be noted on a computerized chart ( Easily) as usual.

Data collection will end 72 hours after the patient's return to the hospital ward. Finally, when a nurse removes the patch, the status of the skin will be assessed: healthy skin (stage 0), redness limited to the contact area between the device and the skin (stage 1), redness extending beyond the contact surface of the device (stage 2), and the appearance of blisters (stage 3). The patients will be asked to evaluate their acceptance of the sensor using a 4-point Likert scale (from 0 = intolerable to 4 = no problem at all). ^11^

Safety assessments will consist of monitoring and recording of all adverse events.

Any participant who wishes to terminate their participation in the study can withdraw from the trial at any time without the need for further explanation. Participants who withdraw from the study will be followed up according to routine clinical practice.

Otherwise, patients will be excluded during the study if they must undergo an unplanned scan or magnetic resonance imaging during the period of monitoring. In this case, the patch will be removed before the radiologic examination.

### Data collection

Demographic data, including age, sex, American Society of Anesthesiologists classification, body mass index, underlying diseases, type and duration of the surgical procedure, and type of surgical procedure, will be collected upon inclusion in the study. Postoperative medications including opioids and other analgesics will also be collected.

All postoperative monitoring variables measured by the nurses (HR, RR, SpO2, SAP, DAP, and temperature) during the first 72 postoperative hours at a frequency determined by the medical indications will be collected using Easily software and recorded on a dedicated electronic case report form (eCRF). The time of occurrence of any complications noted by nurses and doctors will also be recorded on the eCRF.

The wearable device transmits the measurement data every 5 minutes to the Biobeat Gateway through Bluetooth. All data are uploaded to and stored on the Instamed (a French telemedicine company and Biobeat’s partner) certified health data hosting cloud (hosted by Amazon Web Services and General Data Protection Regulation compliant). The research team can then access data through the Instamed platform as well as request that data be exported as.csv files. Nursing staff and doctors will not have access to monitoring data during the protocol duration. The data will be deleted at the end of the study and can be deleted during the study as needed.

### Outcome measures

The main outcome is the occurrence of one or more episodes with a mean arterial pressure below the threshold of 60 mmHg during the first 72 hours following a major non-cardiac surgical procedure unless death or hospital discharge occurs sooner, with the 72-hour period starting when the patient returns to the surgical department. The secondary outcomes will concern the frequency of postoperative respiratory or temperature abnormalities and their duration, the frequency of artefacts and blank/null outputs from the wearable device and, more globally, the signal-level validity, the postoperative complications that occurred during the monitoring period, as determined by the healthcare team in accordance with the Dindo and Clavien classification ^12^ and the comprehensive complication index. ^13^ Finally, the ease of use of the device at the time of insertion and the patient’s tolerance to wearing the Biobeat ^®^ device will also be determined. Definitions of hemodynamic, respiratory, and temperature abnormalities are listed in Table 1.

**Table 1.** Outcome definitions.

Primary Outcome Measure
Postoperative haemodynamic abnormality
• a MAP < 70 mmHg
• or a MAP < 65 mmHg
• or a MAP < 60 mmHg
• or a MAP < 80% of the value measured during the pre-anaesthesia consultation
• or a MAP < 70% of the value measured during the pre-anaesthesia consultation
• or a MAP < 80% of the value measured in the OR before the induction of anaesthesia
• or a MAP < 70% of the MAP measured in the OR before the induction of anaesthesia
• or a MAP > 100 mmHg
• or a MAP > 110 mmHg
• or a MAP > 120 mmHg
• or a MAP > 130 mmHg
• or a MAP > 120% of the MAP measured during the pre-anaesthesia consultation
• or a MAP > 130% of the MAP measured during the pre-anaesthesia consultation
• or a MAP > 120% of the MAP measured in the OR before induction of anaesthesia
• or a MAP > 130% of the MAP measured in the OR before induction of anaesthesia
• or a heart rate < 40/min
• or a heart rate > 100/min
• or a rhythm disorder
Secondary Outcomes
Postoperative respiratory abnormality
• breathing rate < 8/min
• breathing rate > 20/min
• or a peripheral oxygen saturation < 95%
• or a peripheral oxygen saturation ≤ 92%
• or a peripheral oxygen saturation ≤ 90%
• or a peripheral oxygen saturation ≤ 85%
Postoperative temperature abnormality
• a temperature ≤ 36.8°C
• or a temperature ≥ 38°C
• or a temperature ≥ 39°C

MAP: mean arterial pressure; OR.

### Statistics

Sample size

Our main goal is to estimate the proportion of patients showing severe hypotension and, notably, to corroborate the prevalence found in Liem *et al.* ^5^ *i.e.*, 8%. To gain a 10% precision (± 5%) with a two-sided 5% alpha risk, 114 patients need to be included.

Detection of artefacts

This will follow similar rules as previously published in a paper dedicated to the validation a new sensor. ^14^ The rules selected to define artefacts may be updated according to experience and the literature. ^15^ The successive rules will be recorded in a register, and all recordings will be reviewed in the light of these new rules.

Missing values

Missing data will not be replaced.

Statistical analyses

Descriptive summaries will be provided for each parameter and for each device. For continuous variables, the mean, median, and their 95% confidence limits, obtained using bootstrapping methods, will be provided. For discrete variables, counts, percentages, and confidence limits obtained using a bootstrap method will be provided *.*

The proportion of data gaps and artefacts for each parameter will be given as a percentage of the total number of measurement points and observations, respectively, with the corresponding 95% confidence interval (CI).

When physiologic parameters (HR, SAP, DAP, for instance) are measured simultaneously by nurses and wearable devices, a Bland-Altman analysis for repeated measurements, accounting for multiple observations per individual, will be performed to draw mean-difference plots and derive accuracy or bias (mean difference), precision (standard deviation of difference), and limits of agreement (LoAs) that are expected to contain 95% of the paired differences between measurements by the nurses and the wireless patch, with their confidence intervals.

At the patient level and for each kind of clinical event, the proportion of patients with detection of at least one event (hypotension, for instance) by a nurse and wearable monitoring will be described in a two-by-two table. Agreement will be estimated by Cohen’s kappa coefficient with its 95% CI, while differences will be tested by a non-parametric McNemar test for paired nominal data.

The time (hours) to first occurrence of data loss or end of service of the device, or to the first occurrence of a clinical complication, will be described with Kaplan-Meier survival curves, and, if permitted by the number of such events, risk factors will be explored by a Cox model.

Interim analyses are not planned in this study.

A two-tailed p value < 0.05 will be considered statistically significant. All statistical analyses will be performed using R software (R Development Core Team, 2012. https://www.r-project.org/).

### Quality control

Quality control is carried out by the Clinical Research Unit of the sponsor hospital. A qualified person will attest subject eligibility, monitor integrity of the source data and completion of the entries on the electronic case report form, verify the compliance with the clinical study protocol, the Good Clinical Practices, and the regulatory commitments). The monitor will also verify the report of adverse effects.

All subjects will be identified by a unique identification number. Each principal investigator will keep a list in a safe location which will allow the identification of the pseudonymised patients. Patients will be informed about data protection and the fact that data passed to other investigators or an authorized party for analysis will occur in a pseudonymised manner. Data analysis by the biostatistician will also be performed in a pseudonymised manner.

## Discussion

Post-operative monitoring of surgical patients as practiced in general wards is not entirely satisfactory due to its intermittent nature. This monitoring is further degraded in case of reduced nursing staff and during night hours with increased patient/staff ratios. ^16^ This problem is all the more important as the number of patients at risk for postoperative complications will increase, particularly due to the growing population of elderly subjects. ^17^ The possibility of compensating for the lack of monitored beds, whether it is intensive care units and high dependency care units, remote monitoring brings new perspectives. In addition, remote monitoring could be the solution in case of lack of monitored beds.

Leenen *et al.* recently published a systematic review of the literature reporting the feasibility of the use of 13 devices and their validation for in-hospital continuous vital signs monitoring. ^18^ Since this publication, telemonitoring has been studied outside the hospital either in patients who have undergone esophagectomy during the first 7 days at home after discharge ^19^ or in patients with COVID-19. ^9, 20^ Some points remain to be clarified, notably the frequency of artefacts and false alarms and the clinical consequences of the use of remote monitoring.

Our protocol seeks to specify the frequency of artefacts using both automatic detection with predefined bounds but also with review by clinicians as reported in a study concerning the feasibility of continuous monitoring of vital signs in surgical patients on a general ward using SensiumVitals patch. ^21^ This study showed a high percentage of artefacts concerning the respiratory rate measurement (51% of the measurements), and a lower percentage concerning the heart rate measurement (19%) and temperature (9%). This point is crucial as artefacts will generate false alarms leading to the rejection of the technique by caregivers.

We aimed also to measure the gap between the nurse’s observation of an abnormality in a vital parameter and those detected by the remote monitoring. Such an extent will be a strong argument to encourage the generalization of this new type of monitoring, especially as its cost is not yet well known.

This cost has been estimated to be in the order of £400 to £550 per patient in a remote home monitoring model during the COV1D-19 pandemic. ^20^ It will have to be weighed against the cost of a complication avoided or recognized and treated earlier. ^22, 23^

### Limitations

Our protocol suffers from several weaknesses. The first is that this study will take place during the COVID-19 pandemic, which profoundly changes hospital activity and its functioning. In addition, patients included in the study are at intermediate risk since their procedure will last more than 2 hours and they are expected to have a postoperative stay of more than 72 hours but are not expected to be hospitalized in a monitored unit. This obviously reflects a practice specific to each health facility. We decided not to include in the study patients with very hairy thoracic skin since we decided not to ask patients to shave their chests. This induces a bias in the selection of the patients due to the non-inclusion of some men. We chose to have data registered by the nurses gathered at a frequency determined by medical indications and not by a priori determined intervals. Such choice could be considered as a weakness of the protocol, but we wanted to do a real-life study and consequently to have instructions given to nurses by the medical team which may vary from one patient to another. It is of prime importance to notice that only blood pressure measurement passed validation testing for accuracy ^10^ and not the other parameters measured by the Biobeat ^®^ sensor to the best of our knowledge. Although this may be a limitation to this study, we decided to use this device because it received approval from the Food and Drug Administration and European Community regulators. Finally, the number of subjects to be included in the study was based on the results of a previous publication on the occurrence of postoperative hypotension. ^5^ There is probably some difference between our population and our nursing monitoring modalities and the corresponding elements of this study. ^5^ We probably should have done a prior study to better specify the percentage of patients who had a postoperative hypotension in our center. In any case, including 114 patients will allow us to give a first answer to the interest of remote monitoring.

In conclusion, our protocol is specifically aimed to establish whether there is a benefit of remote monitoring using a multiparameter device in the detection of a postoperative complication resulting in an abnormality of one of the major vital signs. This demonstration would encourage the extension of this type of monitoring to hospitalized medical patients and patients at home, which includes many indications: follow-up after discharge, chronic pathology, or acute but not requiring hospitalization.

## Plans for dissemination of the study outcome

Results of the present protocol will be published in peer-review medical journals.

## Data availability

No underlying data are associated with this article but the data for this work will be publicly available in the Dryad repository keeping all data anonymous.
