# Supplementary material for: Screening for postoperative vital signs abnormalities, and particularly hemodynamic ones, by continuous monitoring: protocol for the Biobeat-Postop cohort study
Source: F1000Res. 2021 Oct 7;10:622. Originally published 2021 Jul 21. [Version 2] doi: 10.12688/f1000research.54781.2 (PMC8546735; doi:10.12688/f1000research.54781.2)
Supplement: Peer review history

# Peer review: Reviewer response for version 2

**DOI:** 10.5256/f1000research.77932.r96436

**Published:** 2021-10-25

**Authors:** Zhuo Sun

**Affiliations:** 1 Augusta University, Augusta, GA, USA

I approve of the updated manuscript

Is the study design appropriate for the research question?

Partly

Is the rationale for, and objectives of, the study clearly described?

Yes

Are sufficient details of the methods provided to allow replication by others?

Yes

Are the datasets clearly presented in a useable and accessible format?

Yes

Reviewer Expertise:

Perioperative management, pain management;

I confirm that I have read this submission and believe that I have an appropriate level of expertise to confirm that it is of an acceptable scientific standard.

# Peer review: Reviewer response for version 2

**DOI:** 10.5256/f1000research.77932.r96435

**Published:** 2021-10-22

**Authors:** Philippe Cuvillon

**Affiliations:** 1 Department of Anaesthesiology and Pain Management, Centre Hospitalo-Universitaire (CHU), Nîmes, France

No further comments for this reviewed manuscript.

Is the study design appropriate for the research question?

Yes

Is the rationale for, and objectives of, the study clearly described?

Yes

Are sufficient details of the methods provided to allow replication by others?

No

Are the datasets clearly presented in a useable and accessible format?

Yes

Reviewer Expertise:

Remote monitoring, regional anesthesia

I confirm that I have read this submission and believe that I have an appropriate level of expertise to confirm that it is of an acceptable scientific standard.

# Peer review: Reviewer response for version 1

**DOI:** 10.5256/f1000research.58294.r91571

**Published:** 2021-09-20

**Authors:** Zhuo Sun

**Affiliations:** 1 Augusta University, Augusta, GA, USA

The Authors have submitted a protocol that seeks to quantify the benefit of  remote monitoring every 5 minutes using a multiparametric device in the detection of postoperative complications in comparison with the monitoring performed by nurses. In this proposed study, the authors hypothesize that monitoring with a multi-signal wearable sensor will detect potentially dangerous vital sign abnormalities more often and more rapidly than routine surveillance in surgical patients hospitalized on an unmonitored general care floor. Postoperative hemodynamic abnormalities are common and independently associated with increased postoperative morbidity and mortality. Anesthesiologists are interested in unique methods to safely monitor and manage postoperative patients.

 Strengths: This device looks very user friendly. The enrolled patients will be more likely to adhere to the study compared to other devices used in previous studies.

 Minor concerns:

1. The title is to 'screen for postoperative complications by continuous monitoring'. The primary outcome is the occurrence of one or more hemodynamic complication during the 1 ^st^ 72 hours postoperatively. While in the outcome definition, the primary outcome is defined by different hemodynamic abnormalities. There is no postoperative mortality and morbidity evaluated in this study.
2. Both the occurrence and duration of hemodynamic abnormalities may impact postoperative outcomes. If the author can consider adding the duration of detected hemodynamic abnormalities into the outcomes, this study could provide more information for readers.
3. In the demographic data, the duration of procedure, post-op medications, and opioids/analgesics could be valuable information in the study.
4. The authors hypothesize that monitoring will detect potentially dangerous vital sign abnormalities more often and more rapidly than routine surveillance. It does make sense that continuous monitoring can collect more data, and may detect abnormalities earlier. However, the monitoring data from the patch will be recorded continuously without being communicated to the nursing staff and doctors per protocol. It is unlikely that the abnormal vital signs can be caught rapidly through this study by monitoring.

Is the study design appropriate for the research question?

Partly

Is the rationale for, and objectives of, the study clearly described?

Yes

Are sufficient details of the methods provided to allow replication by others?

Yes

Are the datasets clearly presented in a useable and accessible format?

Yes

Reviewer Expertise:

perioperative management; Pain management;

I confirm that I have read this submission and believe that I have an appropriate level of expertise to confirm that it is of an acceptable scientific standard, however I have significant reservations, as outlined above.

# Response

**Published:** 2021-09-23

**Authors:** Marc Marc

**Affiliations:** Hopital Foch, France

**Responses to Reviewer 2**

 The Authors have submitted a protocol that seeks to quantify the benefit of  remote monitoring every 5 minutes using a multiparametric device in the detection of postoperative complications in comparison with the monitoring performed by nurses. In this proposed study, the authors hypothesize that monitoring with a multi-signal wearable sensor will detect potentially dangerous vital sign abnormalities more often and more rapidly than routine surveillance in surgical patients hospitalized on an unmonitored general care floor. Postoperative hemodynamic abnormalities are common and independently associated with increased postoperative morbidity and mortality. Anesthesiologists are interested in unique methods to safely monitor and manage postoperative patients.

 Strengths: This device looks very user friendly. The enrolled patients will be more likely to adhere to the study compared to other devices used in previous studies.

Minor concerns:

Comment 1: The title is to 'screen for postoperative complications by continuous monitoring'. The primary outcome is the occurrence of one or more hemodynamic complications during the 1st 72 hours postoperatively. While in the outcome definition, the primary outcome is defined by different hemodynamic abnormalities. There is no postoperative mortality and morbidity evaluated in this study.

Response to Comment 1: We agree with this comment and we have changed the title to: “ Screening for postoperative vital signs abnormalities, and particularly hemodynamic ones, by continuous monitoring: protocol for the Biobeat-Postop cohort study”

Comment 2: Both the occurrence and duration of hemodynamic abnormalities may impact postoperative outcomes. If the author can consider adding the duration of detected hemodynamic abnormalities into the outcomes, this study could provide more information for readers.

Response to Comment 2: We agree with this comment, and we have changed the secondary outcomes to: “The secondary outcomes will concern the frequency of postoperative respiratory or temperature abnormalities and their duration, the frequency of artefacts ….”

Comment 3: In the demographic data, the duration of procedure, post-op medications, and opioids/analgesics could be valuable information in the study.

Response to Comment 3: We agree with this comment, and we have changed the first sentence of the Procedure: “Demographic data, including age, sex, American Society of Anesthesiologists classification, body mass index, underlying diseases, type and duration of the surgical procedure, will be collected upon inclusion in the study. Postoperative medications including opioids and other analgesics will also be collected.”

Comment 4: The authors hypothesize that monitoring will detect potentially dangerous vital sign abnormalities more often and more rapidly than routine surveillance. It does make sense that continuous monitoring can collect more data, and may detect abnormalities earlier. However, the monitoring data from the patch will be recorded continuously without being communicated to the nursing staff and doctors per protocol. It is unlikely that the abnormal vital signs can be caught rapidly through this study by monitoring.  

Response to Comment 4: What we seek to measure is the difference between the two monitoring modalities: "classic" monitoring by nurses and "modern" remote monitoring. To do a blind study is mandatory. A significant difference would be an argument for investing in "modern" monitoring. As such, we consider this study to be a pilot study that should provide an initial answer to this question.

# Peer review: Reviewer response for version 1

**DOI:** 10.5256/f1000research.58294.r92786

**Published:** 2021-09-03

**Authors:** Philippe Cuvillon

**Affiliations:** 1 Department of Anaesthesiology and Pain Management, Centre Hospitalo-Universitaire (CHU), Nîmes, France

Thank you for giving me the opportunity to review a topic that is my area of expertise.

 Medical monitoring dedicated to machines or computer systems in health care services will be an issue in the coming years. To date, the proposed systems are disappointing because they provide incomplete or irrelevant data. Monitoring aided by machine learning must offer predictive or immediate detection systems.

 In the proposed study, the authors use a detection system. The main problem of this study is the definition of the primary endpoint, which is incomprehensible (see below)

 Minor remarks:

 Introduction: 

- First paragraph: postoperative mortality and morbidity are time dependent and severity dependent. The authors should specify this temporality (mortality at 30 days?). Similarly, the authors report the danger of hypotension, the studies of which are mainly intra-operative or in continuous care. This should be specified.
- Paragraph 2: specify that to date, no study has been able to demonstrate the real interest of continuous monitoring systems on mortality in health care services, mainly because of automatic monitoring errors.
- Last paragraph: better specify the primary endpoint

Methods:

- Study population: were patients operated on under spinal anaesthesia included? Were patients under epidural or morphine included? These are major biases and independent risk populations for complications (respiratory depression)
- Primary endpoint: very incomplete and not very descriptive "main outcome is the occurrence of one or more hemodynamic complications". Talk about cardiac arrest, drop in blood pressure but in relation to which baseline?
- The NSN depends on the primary endpoint, which is incomplete, which calls into question the NSN

Is the study design appropriate for the research question?

Yes

Is the rationale for, and objectives of, the study clearly described?

Yes

Are sufficient details of the methods provided to allow replication by others?

No

Are the datasets clearly presented in a useable and accessible format?

Yes

Reviewer Expertise:

remote monitoring, regional anesthesia

I confirm that I have read this submission and believe that I have an appropriate level of expertise to confirm that it is of an acceptable scientific standard, however I have significant reservations, as outlined above.

# Response

**Published:** 2021-09-23

**Authors:** Marc Marc

**Affiliations:** Hopital Foch, France

**Responses to Reviewer 1**

 Thank you for giving me the opportunity to review a topic that is my area of expertise.

 Medical monitoring dedicated to machines or computer systems in health care services will be an issue in the coming years. To date, the proposed systems are disappointing because they provide incomplete or irrelevant data. Monitoring aided by machine learning must offer predictive or immediate detection systems

 In the proposed study, the authors use a detection system.

Main remark : The main problem of this study is the definition of the primary endpoint, which is incomprehensible (see below).

Minor remarks:

 INTRODUCTION

Comment 1: Introduction, First paragraph: postoperative mortality and morbidity are time dependent and severity dependent. The authors should specify this temporality (mortality at 30 days?). Similarly, the authors report the danger of hypotension, the studies of which are mainly intra-operative or in continuous care. This should be specified.

Response to Comment 1: The study concerns the early postoperative course (at most 72 first postoperative hours). This is written in the Abstract: “The study will cover the first 72 hours after discharge of the patient from the post-anaesthesia care unit.”  We have changed the Introduction section adding a last sentence to be more precise: “Data obtained using the sensor will be compared to data obtained during the routine nurse follow-up during at most 72 first postoperative hours.” Thus, our study does not include the intraoperative period and the stay in the post anesthesia care unit where patients are continuously under monitoring. Similarly, our study concerns only conventional nursing monitoring performed on  unmonitored general care floors (last paragraph of the Introduction).

Comment 2: Paragraph 2: specify that to date, no study has been able to demonstrate the real interest of continuous monitoring systems on mortality in health care services, mainly because of automatic monitoring errors.

Response to Comment 2: As written in the second sentence of the Introduction, postoperative complications remain quite frequent. We did not want to study postoperative mortality. Moreover, as we are interested in unmonitored general care floors and not  ICU patients, the postoperative risk of death is very low (0.1% in our scheduled surgical population). A study using mortality as main outcome would require a huge number of patients. Otherwise, we agree with the comment about automatic monitoring errors; this is why a large part of the study concerns the screening of artefacts.

Comment 3: Last paragraph: better specify the primary endpoint

Response to Comment 3: We have written in the Outcomes measures section “The main outcome is the occurrence of one or more hemodynamic complications during the first 72 hours following a major non-cardiac surgical procedure unless death or hospital discharge occurs sooner, with the 72-hour period starting when the patient returns to the surgical department.” The main outcome needs effectively to be clarified: “The main outcome is the occurrence of one or more episodes with a mean arterial pressure below the threshold of 60 mmHg during the first 72 hours …..” This definition is in line with the reference used to calculate the number of patients to be included (see Response to comment 6). This threshold has been added to Table 1.

   

 METHODS

Comment 4: Methods: Study population: were patients operated on under spinal anaesthesia included? Were patients under epidural or morphine included? These are major biases and independent risk populations for complications (respiratory depression)

Response to Comment 4: We intend to do this study in real life and locoregional postoperative analgesia is not an exclusion criterion.

Comment 5: Primary endpoint: very incomplete and not very descriptive "main outcome is the occurrence of one or more hemodynamic complications". Talk about cardiac arrest, drop in blood pressure but in relation to which baseline?

Response to Comment 5: See response to Comment 3. As reported in Table 1 (Outcome definitions), abnormalities in mean arterial pressure are defined as absolute values and relative values. Occurrence of a major event, like a cardiac arrest is planned in the Outcome measures section: “… postoperative complications that occurred occur? during the monitoring period, as determined by the healthcare team in accordance with the Dindo and Clavien classification. ^12^”.

Comment 6: The NSN depends on the primary endpoint, which is incomplete, which calls into question the NSN

Response to Comment 6: We have specified the primary endpoint (see response to comment 3). This allows for consistency with the number of subjects to be included which is based on the work of Liem et al.  (Anesthesiology 2020, 133, 510-522) who report that “Postoperative hypotension was common, e.g., 2 cumulative hours below a threshold of 60 mmHg occurred in 144 (8%) patients while 4 h less than 75 mmHg occurred in 824 (48%) patients”. We used this 8% frequency knowing that there is probably some difference between our population and our nursing monitoring modalities and the corresponding elements of the study by Liem et al. We probably should have done a prior study to better specify the percentage of patients who had a postoperative hemodynamic complication requiring a specific treatment. In any case, including 114 patients will allow us to give a first answer to the interest of remote monitoring. We have modified the text to include a limitation of our study: “Finally, the number of subjects to be included in the study was based on the results of a previous publication on the occurrence of postoperative hypotension. ^5^ There is probably some difference between our population and our nursing monitoring modalities and the corresponding elements of this study. ^5^ We probably should have done a prior study to better specify the percentage of patients who had a postoperative hypotension in our center. In any case, including 114 patients will allow us to give a first answer to the interest of remote monitoring.”
